# Analysis of changes in ABT, ABH and ABA after orthodontic treatment of adult bimaxillary protrusion

**DOI:** 10.2340/aos.v84.44123

**Published:** 2025-10-22

**Authors:** Hongrui Sun, Lin Tang

**Affiliations:** Department of Orthodontics, The First Affiliated Hospital of Harbin Medical University, Harbin City, Heilongjiang Province, China

**Keywords:** Orthodontic treatment, bimaxillary protrusion, alveolar bone thickness, alveolar bone area

## Abstract

**Objective:**

To investigate the changes in alveolar bone thickness (ABT), the height of the enamel bone border-alveolar ridge apex (ABH), and alveolar bone area (ABA) of the labial and palatal sides of the maxillary incisor region after orthodontic treatment of adult bimaxillary protrusion.

**Methods:**

Ninety-eight adult patients with bimaxillary protrusion who underwent orthodontic treatment in our hospital between November 2023 and November 2024 were clinically selected for retrospective study, and patient data were obtained from the medical record system, comparing the widths of the intercuspal space between the cuspids, the width of the space between the first premolar teeth, the width of the space between the second premolar teeth, the convexity of the anterior teeth (UI-SN, UI-NA), and the lip and palate of maxillary incisor region before and after treatment. ABT, ABH, and ABA changes.

**Results:**

Comparison of intercuspal width and MP-SN levels before and after treatment in all patients showed no statistically significant differences (*P* > 0.05). The intercuspal width of the first premolar, intercuspal width of the second premolar, UI-SN and MP-SN were higher than those before treatment, and the difference was statistically significant (*P* < 0.05). The labial cervical, labial mid-root, and labial apical ABT levels of maxillary mesial incisors and lateral incisors were higher than those before treatment, and the palatal cervical, palatal mid-root, and palatal apical ABT levels were lower than those before treatment, and the difference was statistically significant (*P* < 0.05). There was no statistically significant difference in the comparison of labial ABH levels of mesial incisors and lateral incisors before and after treatment (*P* > 0.05); the levels of palatal ABH of mesial incisors and palatal ABH of lateral incisors were higher than those before treatment, and the levels of palatal ABA of mesial incisors and palatal ABH of lateral incisors were lower than those before treatment, and the difference was statistically significant (*P* < 0.05).

**Conclusion:**

Orthodontic treatment is effective in adult patients with bimaxillary protrusion, but significant resorption of the alveolar bone on the palatal side of the incisal region occurs after treatment, reducing the amount of alveolar bone in the entire incisal region. Therefore, the long-term effects on periodontal tissues need to be paid attention to.

## Introduction

Bimaxillary protrusion is categorized as simple double arch protrusion or double arch protrusion with abnormal maxillary and mandibular positions, both with medium-sized molar relationships [1]. Clinically, this type of malocclusion is characterized by a protrusion associated with the anterior alveolar segments of the maxilla and mandible, which allows for a protruding or ectropion of the lips, an open-lip, open-toothed, or open-lipped smile, and a small or inconspicuous chin, which ultimately results in a protruding lateral facial profile [2]. This condition is usually due to genetic factors, oral habits (e.g. finger sucking, excessive pacifier use), or other causes [3]. According to the data, the highest rates of bimaxillary protrusion are found in black and yellow populations, lower rates in southern European white and Middle Eastern populations, and the lowest rates in northern European white populations. The prevalence of bimaxillary protrusion in Chinese populations is significantly higher in southerners than in northerners [4]. Bimaxillary protrusion may affect patients’ facial esthetics, masticatory function, and speech articulation [5]. Therefore, the presence of bimaxillary protrusion needs to take effective and reasonable treatment measures in time to correct the malocclusion and avoid its adverse effects.

Along with the gradual development of the economy, and people’s pursuit of their own facial esthetics continues to improve, orthodontic treatment has gradually become the main treatment for patients with bimaxillary protrusion, which has been confirmed to correct the position of the upper and lower jaws, so that the facial contour becomes more coordinated and beautiful, improving the occlusal function and speech pronunciation, and contributing to the patient’s psychological health [6]. The classical orthodontic theory suggests that under the action of appropriate orthodontic force, new bone is deposited on the medial side of the alveolar bone on the tension side, bone resorption occurs on the medial side of the alveolar bone on the pressure side, and at the same time compensatory remodeling occurs on the lateral surface of the cortical bone in order to maintain the original alveolar bone structure and bone volume, that is, tooth movement and alveolar bone remodeling are carried out in the ratio of 1:1 [7]. Obviously, this theory is not perfect, because in clinical practice, alveolar bone resorption due to tooth movement, or even bone windowing and bone cracking, can sometimes be observed. Clinical studies have shown that the incidence of lingual bone dehiscence of maxillary and mandibular anterior teeth increases significantly after orthodontic treatment [8]. In patients with bimaxillary anterior protrusion, extensive movement of the anterior teeth is often required in order to establish normal coverage, which may lead to more medically induced bone damage [9]. Oral and maxillofacial cone beam CT (CBCT) is a new imaging tool used in orthodontics, which is easy to operate, has fast imaging speed, and capable of three-dimensional reconstruction with clear images, which can effectively make up for the defects of the traditional two-dimensional images, and provide strong evidence for orthodontic diagnosis, treatment, and prognostic assessment [10]. CBCT studies have shown that the amount of alveolar bone in the maxillary incisor region of adult patients with Annular Class I bimaxillary protrusion is lower than that of normal control, and treatment options should be considered more carefully during the development of orthodontic plans especially incisor depressions and internal retractions [11]. In adolescent patients with extracted teeth for orthodontic treatment, CBCT showed an increase in alveolar bone thickness (ABT) in the anterior region on the labial side and a decrease in ABT on the lingual side, with the majority of loci showing an increase in ABT with tooth movement [12]. In adult patients, a lack of growth capacity decreases the dynamic remodeling capacity of the alveolar bone, and if orthodontic tooth movement exceeds the remodeling capacity of the alveolar bone, the tooth will likely be displaced out of the alveolar bone, resulting in a lack of bone support for the incisors [13]. Alveolar bone thickness (ABT), enamel bone boundary-alveolar ridge top height (ABH), and alveolar bone area (ABA) are the main parameters of CBCT to assess the changes in the alveolar bone after orthodontic treatment, and there are limited clinical studies on the changes in these parameters after orthodontic treatment in adult patients with bimaxillary protrusion.

Based on this, this study was conducted on adult patients with bimaxillary protrusion who received orthodontic treatment in our hospital. By comparing the changes of ABT, ABH and ABA before and after treatment, it is expected to provide an effective reference for orthodontists to accurately assess the treatment effect on the patients and formulate a more accurate treatment plan, which will, in turn, improve the patients’ masticatory function, facial esthetics, and overall oral health. This will improve patients’ quality of life and promote the development of orthodontic discipline and patients’ oral health.

## Materials and methods

### Statement of ethics

This study was approved by our Institutional Review Board and Ethics Committee. Given that this study was retrospective and only de-identified patient data were used, informed consent was not required as there was no risk or adverse effect on patient care. This waiver is in line with regulatory and ethical guidelines related to retrospective studies.

### Study design

This retrospective analysis studied 98 adult patients with bimaxillary protrusion who underwent orthodontic treatment at our institution between November 2023 and November 2024; all patient data were obtained from the medical record system, and the indicators of the same group of patients before and after treatment were compared without grouping.

### Inclusion criteria

Inclusion criteria included the following: patients with (1) complete CBCT data before and after treatment; (2) bony bimaxillary protrusion, Angle Classification I, receiving orthodontic treatment; (3) age ≥18 years; (4) good oral hygiene; (5) normal cognition and good compliance.

### Exclusion criteria

Exclusion criteria included: patients with (1) dental trauma, pulp necrosis and root canal treatment; (2) severe crowding, dental caries, ambulatory teeth; (3) previous orthodontic treatment history; (4) abnormalities of tooth body, crown-root ratio and root morphology; (5) congenital missing teeth and supernumerary teeth; (6) combined with periodontal diseases, such as periodontitis; (7) X-ray examination showing there were high-density images such as islands in the alveolar bone; (8) combined with systemic diseases; (9) combined with the presence of a number of diseases in the alveolar bone; (10) use of hormone or antibiotic drugs within 3 months before admission; (11) severe facial trauma or deformity; (12) pregnant and lactating women.

### Treatment

CBCT examination was performed before orthodontic treatment and a treatment plan was formulated, and all patients had four first premolar teeth extracted and then bonded brackets (Smartclip, 3M, USA). The maxillary and mandibular second molar teeth were incorporated into the orthodontic system using straight arch orthodontic technology, and the teeth were aligned step by step using nickel-titanium round wires. Before the teeth were aligned, the micro-implant nails (Orthoanchor, Densburg, Germany) were implanted into the patient’s bilateral maxillary and mandibular second premolars and the center of first molar interval at the level of the center point according to the implantation site designed by the preoperative CBCT and the implantation of the stainless steel square wires was used to retract the anterior teeth and elastic skin chains were used to retract the anterior teeth immediately after the implantation. Immediately after implantation, the anterior teeth were retracted with a stainless steel square wire, and the retraction force of the elastic skin chain was 150 g. At the end of the treatment, the fixed orthodontic device and the micro-implant nails were removed, and the CBCT was reviewed and the parameters were measured and compared. All patients were taught oral hygiene before treatment, including the correct method of brushing. The periodontal situation was closely monitored during treatment, and periodontal basic treatment was taken when necessary to prevent gingivitis and periodontitis from occurring and to maximize the protection of periodontal tissue health.

### General data collection

General demographic data of all patients were collected through the medical record system, including age, gender, body mass index (BMI), education level, and orthodontic treatment time.

### CBCT index collection

CBCT was taken with the same machine in all patients before and after treatment, during which the sagittal plane of the patient’s head was perpendicular to the ground, and the Frankfurt Horizontal (FH) plane was parallel to the horizontal plane. Three-dimensional image reconstruction was performed by the NNT software, and the sagittal longitudinal section was adjusted so that it passed through the root apex and was parallel to the tooth’s longitudinal axis of the individual incisors. CBCT was taken with the same machine in all patients before and after treatment, during which the sagittal plane of the patient’s head was perpendicular to the ground, and the FH plane was parallel to the horizontal plane. Three-dimensional image reconstruction was performed by the NNT software, and the sagittal longitudinal section was adjusted so that it passed through the root apex and was parallel to the tooth’s longitudinal axis of the individual incisors. The reconstructed images were imported into ImageJ 2.0 software, and horizontal lines parallel to the FH plane were drawn along the longitudinal axis of the tooth at 3, 6, and 9 mm from the root side of the enamel-osseous junction (CEJ) to represent the root cervical, central, and apical levels of the tooth, respectively, and the following variables were measured: (1) alveolar bone thickness (ABT) of the labial and palatal sides of maxillary incisal region: the alveolar bone thickness of the cervical, central, and apical levels of the roots of maxillary mesial and lateral incisors were measured, respectively; and apical levels of maxillary mesial incisors and lateral incisors, and place a point on the top of the alveolar ridge and the bone cortex, and the software will automatically calculate the distance between the two points, i.e., the thickness of the alveolar bone; (2) area of the alveolar bone of the maxillary incisal region on the lip and palatal side (ABA): manually draw the contours of the alveolar bone of the lip and palatal sides of mesial incisors and lateral incisors, make sure that the contours include the whole area from the top of the alveolar ridge to the bone cortex, and the software will automatically calculate the area of the drawn contour; (3) Height (ABH) of CEJ-alveolar ridge top (ABC) on the labial and palatal sides of maxillary incisal region, i.e., the vertical distance from CEJ to the top of the alveolar ridge is measured; (4) Intercuspal width, intermolar width of the first premolar, intermolar width of the second premolar, and anterior camber: ① Intercuspal width: a point is placed in the proximal-medial contact point of the corresponding cuspid on the maxilla or the mandible, another point is placed in the distal-medial contact point of the cusp on the other side. Another point is placed at the distal contact point of the other side of the cusp, and the distance between the two points is measured as the width between the cusps; ② Inter-cusp width of the first premolar: place a measuring point at the proximal contact point of the first premolar and the distal contact point of the first premolar on the opposite side of the cusp, and measure the distance; ③ Inter-cusp width of the second premolar: place a measuring point at the proximal contact point of the second premolar and the distal contact point of the second premolar on the opposite side of the cusp, and measure the distance; ④ Anterior Convexity: Upper Intermediate Incisor angle (UI-SN): connect the upper mesial incisor and the butterfly saddle point, and then connect the butterfly saddle point with the root point of the nose, and measure the angle between the two lines; vertical distance from the incisal end of the upper mesial incisor to the line of NA (UI-NA): vertical distance between the line of the upper mesial incisor to the root point of the nose and the line of the upper alveolar seat point to the root point of the nose); angle of the mandibular plane (MP-SN): choose the intersection point between the mandibular plane and the cranial plane, and measure the distance between these two lines; angle of the mandibular plane (MP-SN): select the intersection point of the mandibular plane and the cranial base plane, and measure the angle between these two lines.

### Statistical analysis

SPSS25.0 statistical software was used to analyze the data, and the measurements that conformed to normal distribution were expressed as (x̄ ± s). The paired-sample t-test was taken before and after treatment within the group. The difference was considered statistically significant at *P* < 0.05.

## Results

### Baseline information

Among the 98 adult patients with bimaxillary protrusion, there were 30 males and 68 females; ages ranged from 18 to 28 years old, with a mean value of (23.40 ± 2.58) years; BMI ranged from 18.9 to 22.9 kg/m^2^, with a mean value of (20.32 ± 1.20) kg/m^2^; education level was 39 cases in junior high school and below, and 59 cases in senior high school and above; and the time of orthodontic treatment ranged from 2 to 3 years, with a mean value of (2.20 ± 0.48) years.

### Comparison of intercuspal width, first premolar width, second premolar width, UI-SN, UI-NA and MP-SN before and after treatment in all patients

Intercuspal width, first premolar width, second premolar width, UI-SN, UI-NA and MP-SN were used to evaluate the effect of orthodontic treatment. The greater the increase in each index after treatment, the better the effect of orthodontic treatment. Comparing intercuspal width and MP-SN levels before and after treatment, the differences were not statistically significant (*P* > 0.05) (see Table 1).

**Table 1:** Comparison of intercuspal width and MP-SN before and after treatment in all patients (x̄ ± s, *n* = 98).

Index	Prior-treatment	Pre-treatment	*t*	*P*
Intercuspal width (mm)	33.02 ± 4.50	32.68 ± 3.58	0.585	0.559
MP-SN(°)	31.20 ± 5.12	31.58 ± 5.34	0.508	0.612

Note: MP-SN is mandibular plane angle.

**Figure 1:**
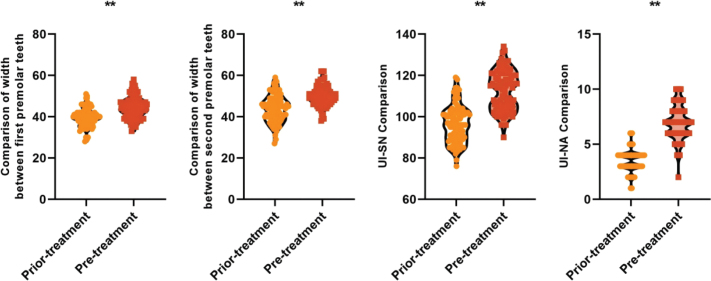
Comparison of first premolar intermolar width, second premolar intermolar width, UI-SN and UI-NA before and after treatment in all patients Note: UI-SN is the upper mesial incisor angle; UI-NA is the perpendicular distance from the incisal end of the upper mesial incisors to the line connecting them to the NA; ** is P < 0.001.

After treatment, the intermolar width of the first premolar (44.36 ± 5.22 vs. 39.57 ± 4.45) mm, the intermolar width of the second premolar (49.88 ± 4.76 vs. 43.85 ± 6.48) mm, the UI-SN (112.64 ± 9.57 vs. 96.39 ± 8.92)°, the UI-NA (6.95 ± 1.50 vs. 3.48 ± 1.02)° were higher than before treatment, and the difference was statistically significant (*P* < 0.05), thus indicating that orthodontic treatment of adult bimaxillary protrusion can achieve good efficacy (see Figure 1).

### Comparison of labial and palatal ABT of maxillary mesial incisors before and after treatment in all patients

**Figure 2:**
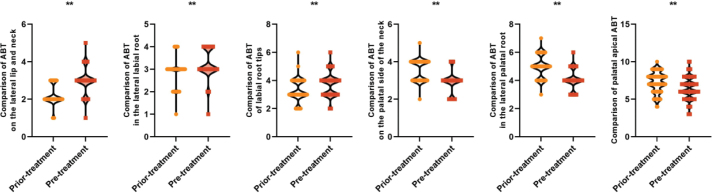
Comparison of labial and palatal ABT of maxillary mesial incisors before and after treatment in all patients Note: ABT is alveolar bone thickness; ** is P < 0.001.

**Figure 3:**
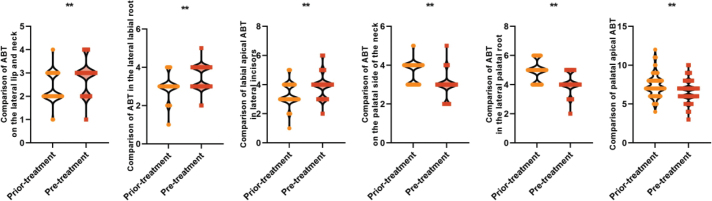
Comparison of labial and palatal ABT of maxillary lateral incisors before and after treatment in all patients Note: ABT is alveolar bone thickness; ** is P < 0.001.

ABT level reflects the alveolar bone changes after orthodontic treatment, and the lower ABT level represents the more severe alveolar bone resorption. All patients had higher ABT levels in the labial cervical (2.95 ± 0.65 vs. 2.20 ± 0.54) mm, labial mid-root (3.25 ± 0.62 vs. 2.87 ± 0.63) mm, and labial apical (3.68 ± 0.78 vs. 3.26 ± 0.75) mm ABTs of their maxillary mesial incisors after treatment than before, and in the palatal cervical (2.88 ± 0.56 vs. 3.54 ± 0.50) mm, palatal lateral root mesial (4.02 ± 0.69 vs. 4.98 ± 0.76) mm, and palatal lateral apical (6.44 ± 1.40 vs. 7.21 ± 1.35) mm ABT levels were lower than those before treatment, and the difference was statistically significant (*P* < 0.05). All patients had higher levels of labial cervical (2.85 ± 0.60 vs. 2.37 ± 0.58) mm, labial mid-root (3.48 ± 0.57 vs. 2.96 ± 0.62) mm, and labial apical (3.89 ± 0.76 vs. 3.35 ± 0.72) mm ABT in the maxillary lateral incisors and higher levels of palatal cervical (2.92 ± 0.58 vs. 3.65 ± 0.54) mm, palatal root mesial (4.12 ± 0.65 vs. 4.90 ± 0.69) mm, and palatal apical (6.65 ± 1.30 vs. 7.32 ± 1.42) mm ABT levels were lower than those before treatment, and the difference was statistically significant (*P* < 0.05). This suggests that the palatal ABT after orthodontic treatment of adults with bimaxillary protrusion shows a decreasing trend, and alveolar bone resorption exists on the palatal side (see Figures 2 and 3).

### Comparison of ABH before and after treatment in all patients

ABH can be used to assess alveolar bone changes after orthodontic treatment, and higher ABH levels represent more severe alveolar bone resorption. The difference in ABH levels of mesial incisors labial and lateral incisors labial before and after the treatment of all the patients was not statistically significant (*P* > 0.05) (see Table 2).

**Table 2:** Comparison of labial ABH before and after treatment in all patients (x̄ ± s, *n* = 98).

Index	Prior-treatment	Pre-treatment	*t*	*P*
Labial side of the middle incisor(mm)	2.35 ± 0.62	2.29 ± 0.58	0.700	0.485
Lateral incisor labial(mm)	2.27 ± 0.57	2.32 ± 0.60	0.598	0.550

Note: ABH is the height of the enamel bone boundary-top of the alveolar ridge.

**Figure 4:**
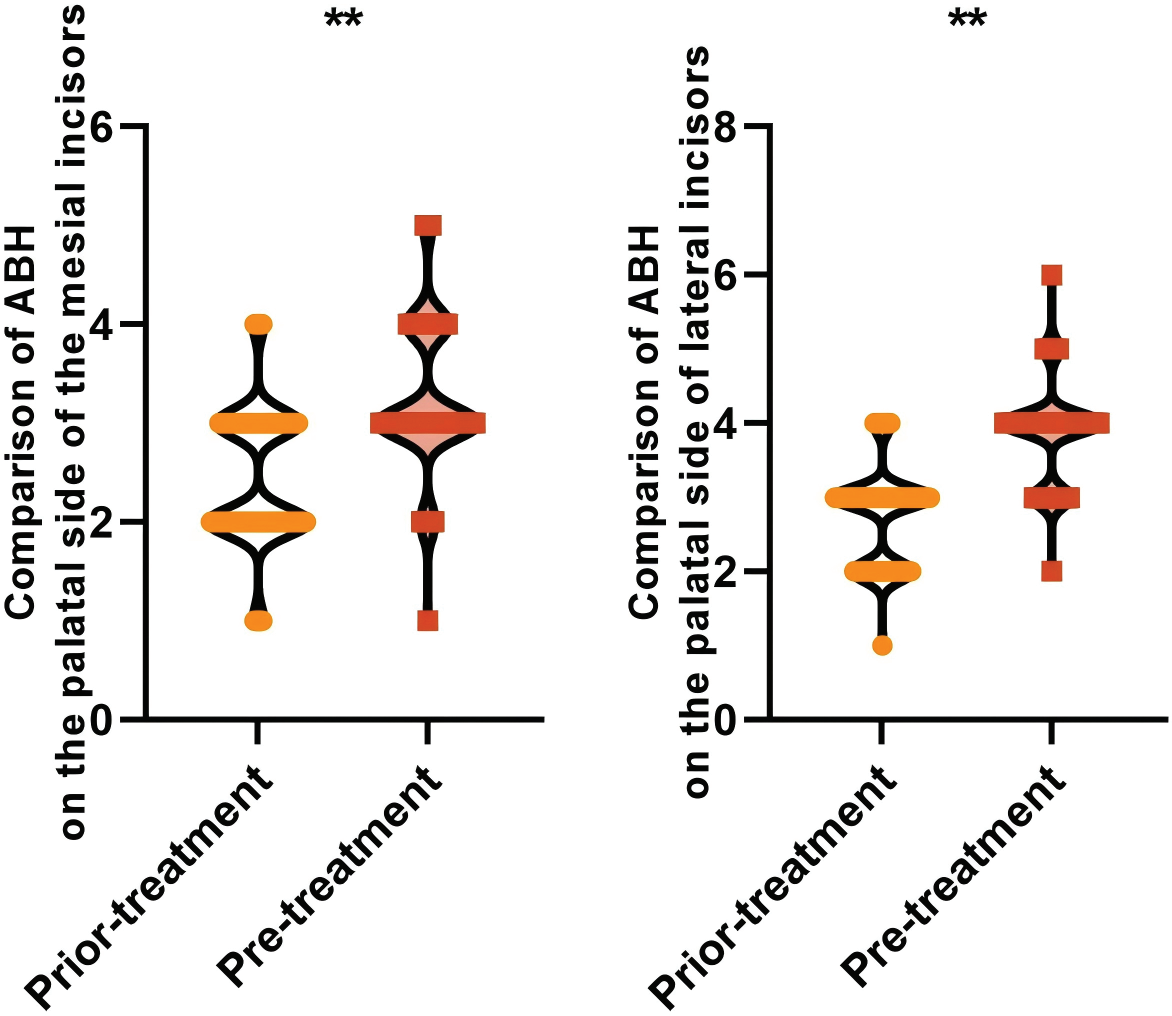
Comparison of palatal ABH before and after treatment in all patients Note: ABH is the height of the enamel bone boundary-top of the alveolar ridge; ** is P < 0.001.

The ABH levels on the palatal side of the mesial incisors (3.25 ± 0.74 vs. 2.46 ± 0.68) mm and the palatal side of the lateral incisors (3.86 ± 0.64 vs. 2.70 ± 0.65) mm were higher than those before the treatment after treatment, and the difference was statistically significant (*P* < 0.05), which suggests that the palatal side of the ABH showed a tendency to be elevated after orthodontic treatment of adult bimaxillary protrusion, and that there was a more pronounced palatal side of the bone resorption (see Figure 4).

### Comparison of ABA before and after treatment in all patients

ABA can also be used to assess alveolar bone changes after orthodontic treatment, with lower ABA levels representing less alveolar bone volume. There was no statistically significant difference in the comparison of ABA levels before and after treatment in all patients for mesial incisors (labial) and lateral incisors (labial) (*P* > 0.05), as shown in Table 3.

**Table 3:** Comparison of labial ABA before and after treatment in all patients (x̄ ± s, *n* = 98).

Index	Prior-treatment	Pre-treatment	*t*	*P*
Labial side of the middle incisor (mm^2)^	8.74 ± 1.55	8.98 ± 1.64	1.053	0.294
Lateral incisor labial (mm^2)^	6.95 ± 1.42	7.12 ± 1.56	0.798	0.426

Note: ABA is alveolar bone area.

**Figure 5:**
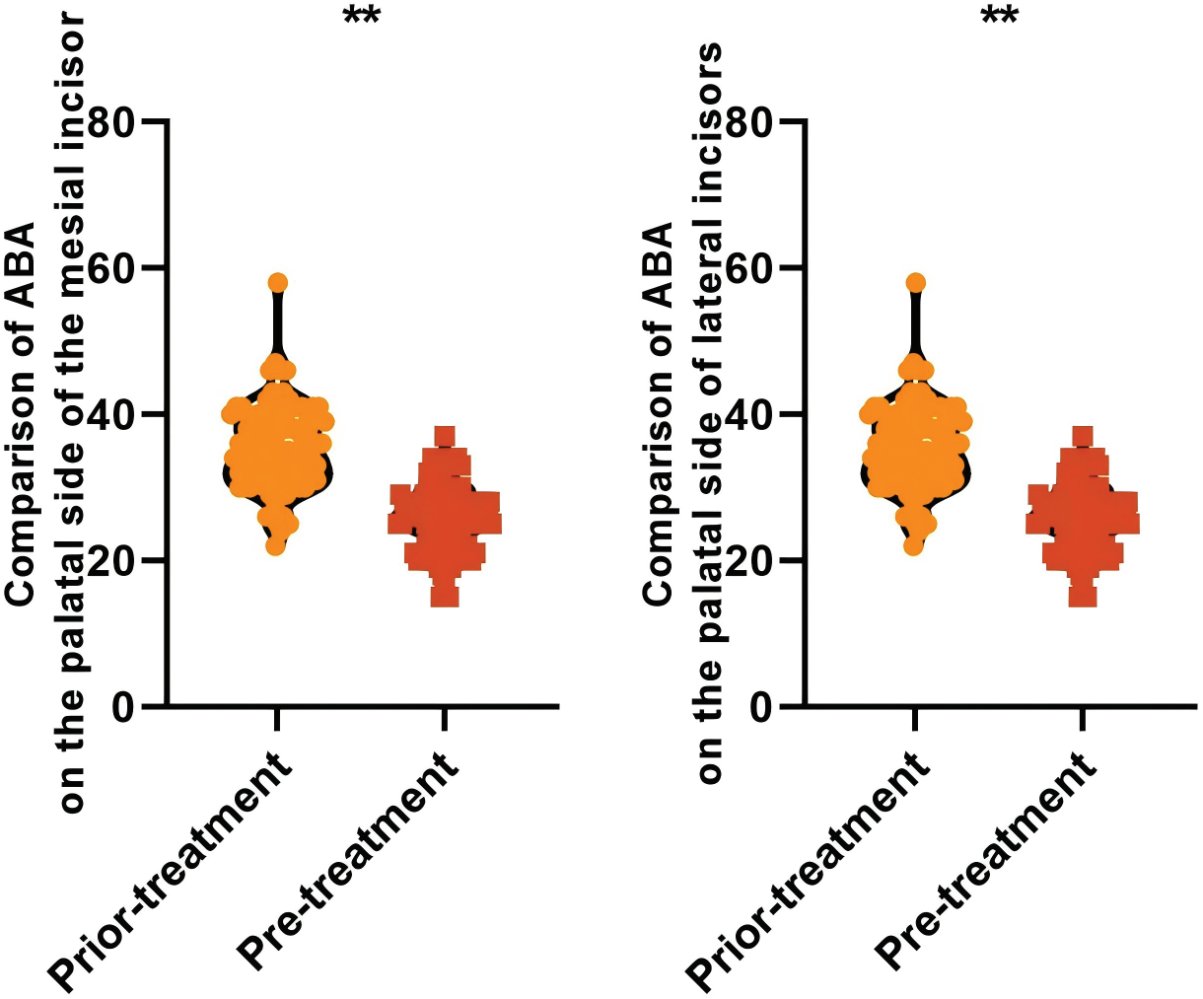
Comparison of ABA on the palatal side before and after treatment in all patients Note: ABA is alveolar bone area; ** is P < 0.001.

The ABA levels on the palatal side of the mesial incisors (25.84 ± 4.32 vs. 35.26 ± 5.80) mm^2^ and on the palatal side of the lateral incisors (19.70 ± 3.30 vs. 28.44 ± 4.32) mm^2^ were lower after treatment than those before the treatment, and the difference was statistically significant (*P* < 0.05), thus indicating that the palatal side of ABA showed a tendency to be reduced after orthodontic treatment of adult bimaxillary protrusion. There was a significant reduction in the amount of alveolar bone (see Figure 5).

## Discussion

Bimaxillary protrusion is a type of malocclusion characterized by lip tilt and lip protrusion of the upper and lower anterior teeth, and is a common malocclusion in East Asia [14]. The main goal of such patients seeking orthodontic treatment is to improve the negative impact of protruding teeth and lips on their appearance, and treatment often requires extraction of four premolar teeth followed by substantial retraction of the incisors to achieve a better improvement in lateral appearance [15]. In this study, we found that the width between the first and second premolar and the convexity of the anterior teeth improved after treatment, and there was significant alveolar bone resorption on the palatal side of the mesial and lateral incisors, which suggests that orthodontic treatment is effective, but the degree of attention to alveolar bone resorption needs to be increased after treatment.

The first premolar intermolar width, second premolar intermolar width, UI-SN, and MP-SN are commonly used indicators for clinical evaluation of orthodontic outcomes in patients with bimaxillary protrusion. In the present study, we found that the first premolar intermolar width, second premolar intermolar width, UI-SN and MP-SN were higher than those before treatment in all patients, suggesting that orthodontic treatment has an orthodontic effect on adult bimaxillary protrusion patients. The study of Chung et al. [16] found that extraction orthodontic treatment can effectively improve the convex facial shape and improve the anterior convexity of the patients with bimaxillary protrusion with a good orthodontic effect, which is consistent with the results of this study. The orthodontic treatment of bimaxillary protruding patients in this study adopts the implant nail support force, and the orthodontic force is located in front of the impedance center of the teeth, with light force and small friction, and the flexible and suitable orthodontic force is conducive to the alignment of the teeth faster and promotes the expansion of posterior arches, and the alveolar bone tissue undergoes adaptive modification to provide gaps for the protruding teeth to be aligned, and enhances orthodontic effect, which in turn promotes the width of the first premolar, the width of the second premolar, and the width of UI-SN and the width of the second premolar, and improves the orthodontic effect. width, UI-SN and MP-SN improvement [17, 18].

In patients with bimaxillary protrusion, if the incisors are excessively retracted during orthodontic treatment, the excessive force may cause the roots to break through the ‘anatomical limitations’ of the alveolar bone and contact the bone cortex of the alveolar bone, resulting in alveolar bone resorption and complications such as bone opening and cracking, which may affect the gingival attachment and jeopardize periodontal health [19, 20]. Excessive internalization of the anterior teeth is more likely to lead to a reduction in the thickness of the alveolar bone in patients with thin cortical bone [21, 22]. The pattern of alveolar bone remodeling during orthodontic tooth movement has been controversial. Ideally, when the teeth are subjected to appropriate orthodontic forces, the alveolar bone undergoes a remodeling process of ‘bone resorption on the pressure side and bone deposition on the tension side’, which is called ‘bone movement’ [23, 24]. However, the results of a CBCT study showed that in adult patients, tooth movement may be ‘bone-through’, with no significant alveolar bone regeneration after tooth movement, and the root tip is in contact with the palatal bone cortex [25]. The results of this study showed that although the labial side of the maxillary incisors increased, the ABT and ABA of the palatal side of the upper anterior teeth decreased, while the ABH increased, suggesting that during orthodontic tooth movement in adult patients, the teeth move ‘through the bone’ rather than ‘with the bone’, which suggests that the orthodontist may move the teeth ‘through the bone’. This suggests that orthodontists should take the local anatomical characteristics of the alveolar bone, orthodontic tooth movement patterns, and periodontal remodeling potential into consideration when planning treatment for patients with bimaxillary protrusion.

In this study, alveolar bone changes in the maxillary incisor region were measured and studied by CBCT, and resorption was found to have occurred on the palatal side of the maxillary incisors at three levels: the root cervical, mid-root, and apical levels, which suggests that orthodontic treatment can affect the periodontal supporting tissues on the palatal side. Although the thick mucosa on the palatal side of the maxillary anterior region has a strong resistance to bacterial invasion, once the patient develops inflammation of the periodontal tissues, it will likely progress rapidly; moreover, once the patient needs to be extracted for implant restorations due to severe caries or traumatic injuries, the alveolar bone after orthodontic treatment will pose a challenge to the implantologist [26, 27]. Therefore, when orthodontists make treatment plans for patients with bimaxillary protrusion, they need to consider not only the distance and manner of tooth movement, but also the impact of orthodontic treatment on the alveolar bone. It is also important to note that biological factors, that is, the patient’s age and the condition of his or her own alveolar bone, also need to be considered during orthodontic tooth movement [28, 29]. A study by Kobylyanskyy et al. [30] found that in adolescents who underwent extraction treatment, the alveolar bone in the anterior region generally showed an increase in the thickness of the alveolar bone on the labial side, and a decrease in the alveolar bone on the lingual side, and the anterior teeth showed a reduction of the ABT after the inward retraction of the teeth in most of the loci. Most of the sites showed an increase in ABT with tooth movement. This suggests that adolescents may have a greater remodeling capacity than adults, with ‘bone-on-bone’ movement; however, the above study did not differentiate between cases of crowding or bimaxillary protrusion. In our study, we selected adult patients with bimaxillary protrusion undergoing orthodontic treatment, and the inclusion criteria of ≥18 years of age only minimally excluded the influence of growth; given the potential radioactivity of CBCT, there are no reports confirming whether adolescents do indeed show ‘banded’ bone movement.

However, there are still limitations in this paper: (1) This study is a single-center retrospective analysis relying on existing clinical databases, which may lead to incomplete data recording or measurement bias. Although we controlled errors through standardized CBCT measurement procedures (e.g. the same equipment, fixed operators), the retrospective design cannot completely avoid selection bias (e.g. only including patients who completed their full treatment). Future prospective studies are needed to further validate our results. (2) This study included 98 patients, which is one of the larger single-center studies in this category. According to preliminary power calculations (based on pre-experiment effect sizes), the sample size meets the requirements for statistical power (>0.8) for the primary outcomes (e.g. changes in ABT). However, there are still limitations due to regional and population constraints. Multi-center large-sample studies can further enhance the generalizability of the results. (3) This study only compared immediate pre- and post-treatment CBCT data and lacks long-term follow-ups exceeding 5 years. Alveolar bone remodeling is a dynamic process, especially in adult patients with weaker periodontal tissue repair capabilities. Short-term data cannot fully reflect the long-term effects of orthodontic treatment on alveolar bone (e.g. whether palatal bone resorption worsens over time). Future studies need to conduct follow-ups exceeding 5 years and combine periodontal indices (e.g. attachment loss, annual changes in alveolar bone height) to evaluate treatment safety and stability.

## Conclusion

In summary, orthodontic treatment for adult patients with bimaxillary protrusion is effective, but significant resorption of the alveolar bone on the palatal side of the incisal region occurs post-treatment, resulting in a decrease in the amount of alveolar bone in the entire incisal region. To address these challenges, future research could focus on: (1) developing biomechanical strategies (e.g. low-force orthodontics or customized appliance design) to minimize palatal bone resorption; (2) exploring biological modifiers (e.g. bisphosphonates or growth factors) to promote alveolar bone remodeling during tooth movement; (3) conducting long-term multicenter studies to validate the stability of alveolar bone changes and their clinical implications for periodontal health and implant dentistry.

## Data Availability

The datasets used and/or analyzed during the current study are available from the corresponding author on reasonable request.
